# Knowledge, Attitudes, and Practices of Primary Care Physicians towards COVID-19 in Greece: A Cross-Sectional Study

**DOI:** 10.3390/healthcare10030545

**Published:** 2022-03-16

**Authors:** Emmanouil K. Symvoulakis, Ioannis Karageorgiou, Manolis Linardakis, Dimitrios Papagiannis, Chrissi Hatzoglou, Aristotelis Symeonidis, Georgios Rachiotis

**Affiliations:** 1 Clinic of Social and Family Medicine, Department of Social Medicine, Faculty of Medicine, University of Crete, 71003 Heraklion, Greece; linman@med.uoc.gr; 2 School of Medicine, University of Crete, 71003 Heraklion, Greece; ionkarag@gmail.com; 3 Public Health & Vaccines Lab., Department of Nursing, School of Health Sciences, University of Thessaly, 41110 Larisa, Greece; dpapajon@gmail.com; 4 Department of Physiology, Faculty of Medicine, University of Thessaly, 41500 Larissa, Greece; chatz@med.uth.gr; 5 Bioinformatics and Human Electrophysiology Laboratory, Department of Informatics, Ionian University, 49100 Corfu, Greece; arisymeon97@gmail.com; 6 Department of Hygiene and Epidemiology, Faculty of Medicine, University of Thessaly, 41500 Larissa, Greece; g.rachiotis@gmail.com

**Keywords:** COVID-19, knowledge, attitudes, practices, primary care, vaccine

## Abstract

The aim of this study was to investigate the knowledge, attitudes, and practices of primary care physicians and residents towards the COVID-19 pandemic in Greece. A cross-sectional questionnaire-based study was conducted in Greece during March 2021. The population frame for the study was a list of currently practicing primary care physicians and residents who were registered within one of the main associations of general/family medicine in Greece. Hierarchical multiple logistic regression analysis was performed for practices at higher levels (vs. lower) in relation to knowledge, attitudes, and general characteristics of participants. Overall, 194 participants completed the survey (e-response rate: 38.4%). In total, 94% of participants were familiar with official recommendations regarding SARS-CoV-2, and 88.7% were vaccinated against SARS-CoV-2 or promptly intended to be. Physicians working in the private sector had a higher average practices score when compared to physicians working in the public sector (87.6 vs. 81.9, *p* < 0.05). Higher levels of attitudes predicted greater odds for higher levels of practices (odds ratio = 4.18, *p* < 0.05). Despite the relatively high COVID-19 vaccination rate of physicians, several participants were unvaccinated due to a then unscheduled first dose appointment. Attitudes were the only determinant for more proper practices towards the prevention of COVID-19.

## 1. Introduction

SARS-CoV-2, the virus known to cause COVID-19, is a positive-sense, single-stranded RNA virus belonging to the family of coronaviruses [1]. Coronaviruses can infect humans and many animals and cause a variety of diseases [2]. There have been outbreaks of coronavirus infections in the past, namely SARS (2002) [3] and MERS (2012) [4]. SARS-CoV-2 was first identified as the cause of a respiratory disease affecting people in Wuhan, China [5]. In the following weeks from the first case reported, the virus spread rapidly throughout China and then worldwide [6], which led to WHO declaring the SARS-CoV-2 infection a pandemic on 11 March 2020 [7]. Since then, there have been 430,257,564 confirmed cases and 5,922,047 deaths from the disease across the world [8] (from the start of the pandemic until 25 February 2022). The first case of COVID-19 in Greece was confirmed on 20 February 2020. Since then, there have been a total of 2,369,396 confirmed cases of COVID-19 with 25,603 deaths reported to WHO [9] (from the start of the pandemic until 25 February 2022). Healthcare professionals, who were at the frontlines since the beginning of the pandemic, were at increased risk of contracting the virus [10]. Proper use of personal protective equipment (PPE) has been proven to reduce infection rates of healthcare workers [11], even though the ideal type of protection has been an issue of extensive discussion [11], and training on how to use the equipment is required [12]. Moreover, the risk of infection can be reduced by adequate knowledge, a positive attitude, and correct practices [13,14]. So far, several studies of knowledge, attitudes, and practices (KAP) have been conducted worldwide, both in the general population and among healthcare professionals [13,15,16,17]. Several studies have found that, among the general population, males, younger persons, people with less education, and people belonging to ethnic minorities have lower average KAP scores [18,19]. These figures regarding knowledge among health groups can show more positive trends, with appropriate education tailored to the needs of individuals and communities [20]. Globally, lower income population groups tend to have lower scores in comparison to high-income groups [18]. Some KAP studies have also been conducted in Greece, in both community groups and healthcare workers, with their findings in accordance with the international literature findings [21,22]. This study was conducted during March 2021, a critical and early time period for the vaccination campaign in Greece, a year after the first lockdown [23]. Vaccine availability for all brought a spirit of collective optimism and especially for physicians and front-line healthcare workers, already heavily burdened, due to their escalating duties and responsibilities. The vaccine was seen as a hope for returning to the vanished routine, and as an “antidote” to the fear of infection, stigma, hospitalization, or death. This period cultivated mixed feelings of uncertainty and hope. However, vaccination was not unanimously positively seen as an option, and many global population studies had already documented this, even before the vaccine became available [24]. Higher income countries and citizens reporting trust in the government’s recommendations were more likely to accept the vaccine [24]. Among the high-income countries, commonly reported reasons for denying vaccination were issues regarding vaccine safety and efficacy, the perception of being at low risk of severe infection, and low levels of trust in state authorities or other institutions [25]. Significant heterogeneity regarding vaccine acceptance was seen among health workers from countries with high-income versus low- to middle-income countries, with the former reporting a higher propensity to accept the vaccine [26].

Since the beginning of the vaccination campaign, vaccine hesitancy has been a reason for public discussion and social reactions for many countries globally and especially across Europe [27]. Therefore, many European countries made decisions about vaccine incentives for compliant groups, in the form of green passes, or penalties for non-compliant ones, such as fines for non-vaccination [28]. This sparked deviating opinions, with many people rejecting the option of mandatory vaccination, regardless of their vaccination status [29].

Even though primary care should be the access point of the healthcare system in the management of the pandemic, and primary care physicians (PCPs) are considered the coordinators of care in this setting, studies, both national and international, examining the knowledge, attitudes, and practices of this group of healthcare professionals are limited. Moreover, the vaccination of primary care doctors is of utmost importance because PCPs are a source of important information regarding disease prevention. However, there is a limited number of studies at a national level and an early vaccination coverage phase for primary care physicians exclusively. This study aims to investigate the knowledge, attitudes, and practices of primary care physicians towards COVID-19 in Greece.

## 2. Materials and Methods

### 2.1. Study Design, Sample, and Participants

A cross-sectional questionnaire-based study was conducted in Greece. The population frame for the study was a list of currently practicing physicians and residents in primary care, who were registered at Hippocrates General and Family Medicine Association, Greece. The questionnaire was sent to the listed email addresses through an emailing service application. Respondents’ answers to the questionnaire items were sent as encrypted information. Replies to the questionnaire were accepted within one month of its delivery date (March 2021). Due to the low response rate related to studies involving PCPs [30,31,32], we aimed to capture, as respondents, a third from a list of 1580 email addresses that Hippocrates Associations holds and uses for scientific purposes (from those about 1100 are members). In 2020, 3465 PCPs were active at a national level [33].

### 2.2. Research Tool

The questionnaire used to collect relevant information contained three sections (knowledge section, attitudes section, practices section) plus general socio-demographic information, as is the case with other KAP studies in the literature [21]. The 3 sections consisted of 9, 5, and 10 questions, respectively, for a total of 24 questions. In the knowledge section, for five of the questions, a five-point scale was used for answering (fully disagree/disagree/agree/fully agree/do not know), while for the remaining four, a trichotomized response was used (yes/no/do not know). The correct answers to the questions in the knowledge section were derived after consensus from guiding information and reports periodically published by international health care authorities [34]. In the attitudes section, three of the questions were answered using a five-point scale (always/most of the time/sometimes/less of the time/never) while the remaining two were answered using a dichotomized response (yes/no). In the practices section, seven of the questions were answered using the above-mentioned dichotomized manner, two of the questions used a different five-point scale (before and after contact with patients/before performing sterile procedures/after exposure to patient biological fluids/after patient contact/after contact with the patient’s surroundings) while the last question used the five-point scale described in the attitudes section. The responses of the participants were then scored as follows: in the knowledge section, correct answers were given one point while incorrect answers were given 0; in the attitudes section, actions or concerns aimed towards the prevention of SARS-CoV-2 were given one point; and finally, practices that were performed for the prevention of disease transmission were given one point. The score for each category was the sum of the total points acquired in that category and was calculated separately for each of the 3 categories and was then converted to a scale of 0–100. Higher score levels were considered those at or above the 75th percentile (4th quartile on the scale of 0–100).

### 2.3. Statistical Analysis

Data were analyzed using SPSS software (IBM SPSS Statistics for Windows, Version 26.0. Armonk, NY, USA: IBM Corporation). The distributions of the general characteristics of the 194 primary care physicians and residents were estimated. The 95% confidence intervals (95% CIs) were also estimated for the right/positive responses in each of the 3 sections comparing their distributions [35]. Score levels of the sections were also estimated, and their distribution was tested for symmetry using Blom’s method (QQ plot). As they were found to have a non-normal distribution, their levels were compared between the vaccination status (yes/no) or general characteristics categories with the Mann–Whitney and Kruskal–Wallis methods [35]. To assess the effect sizes in comparisons, the r effect size index based on z-statistic was estimated. Finally, hierarchical multiple logistic regression analysis was implemented for practices’ higher levels (vs. lower) in relation to the knowledge, attitudes, and general characteristics of participants. The critical value was set at 0.05 [35].

## 3. Results

### 3.1. General Characteristics of Healthcare Professionals

We can estimate that we collected information from more than 5% of PCPs working in the country. Automated metric feedback confirmed access to the informative email with the linked questionnaire from 504 receivers. Replies accounted for 38.4% (n = 194).

Of the 194 primary care physicians and residents that submitted their survey, 42.3% were men (Table 1), the mean age of participants was 46.5 years, and 29.4% of physicians were 50 years old or older. The median years from residency completion was 9.0, and 37.0% of physicians declared that they had a Ph.D./MSc degree. The most common workplaces reported were health centers (n = 70; 36.1%), the private sector (n = 51; 26.3%), and hospitals (n = 40; 20.6%).

### 3.2. Knowledge, Attitudes, and Practices

The positive responses of the participants to the questions posed to them to the questionnaire’s three categories are shown in Table 2. Significant findings, among others, were the following:

In the knowledge section, a very high percentage of participants were aware of the recommendations regarding SARS-CoV-2 (93.8%; 95%CI 89.8, 96.6). When it comes to the treatment of COVID-19, a small percentage of physicians responded correctly (specific treatment available: 10.8%; 95%CI 7.0, 15.8, antibiotics useful for treatment: 21.6%; 95%CI 16.3, 27.8). Similarly, low correct response rates were seen for questions regarding the mode of transmission of SARS-CoV-2 (transmitted by consuming food: 15.5%; 95%CI 10.9, 21.0). In the attitudes section, a significant portion of the participants were vaccinated against SARS-CoV-2 or intended to promptly be (88.7%; 95%CI 83.6, 92.5). Moreover, it is noteworthy that 10.8% (n = 21) of participants had already contracted COVID-19, as shown on Table 1.

In the practices section, it showed that many participants did not make proper use of a facemask, despite being aware of the recommendations by WHO. More specifically, only 77.8% (95%CI 71.6, 83.2) did not touch the facemask while wearing it, and 59.3% (95%CI 52.3, 66) did not reuse their mask during the day. Proper disposal of the facemask was performed by 69.6% (95%CI 62.9, 75.7) of primary care physicians. However, a significant percentage of participants followed appropriate hygiene practices after touching the facemask (93.3%; 95%CI 89.1, 96.2).

The score levels on the three categories of the questionnaire are shown in Table 3. The scores were transformed to a homogeneous scale (0–100), where higher values indicate better knowledge, more positive attitudes, or correct practices. Knowledge levels are estimated to be lower on average compared to attitudes and practices levels (49.0, 94.1, and 83.4 respectively), whereas only 7.7% of participants were found to have high (score 75.0+) knowledge levels. On the contrary, higher score levels were seen for attitudes (94.3%) and practices (71.1%).

No significant differences were found in the score levels of knowledge, attitudes, and daily practices of the 194 participants when assessed according to gender, age, years from residency completion, and postgraduate degree (results not shown in table/figure). However, significant differences were found in the above-mentioned scores of participants depending on the workplace (public vs. private sector) and COVID-19 infection status. Firstly, physicians working in the private sector seemed to have a higher average practices score when compared to physicians working in the public sector (U-test: (87.6 (±15.2) vs. 81.9 (±18.5), *p* < 0.05; z = −1.893, r effect size = 0.27)). Secondly, participants who contracted COVID-19 seemed to have significantly lower average knowledge scores (U-test: (39.7 (±13.4) vs. 50.2 (±17.1), *p* < 0.05; z = −2.853, r effect size = 0.62)) Based on the hierarchical multiple logistic regression analysis seen in Table 4, the higher levels of practices, in relation to counterparts, seem to not be significantly related with the general characteristics (model 1) or/and knowledge higher levels (model 2). Nevertheless, attitudes’ higher levels (model 3) seem to define a greater odds for higher levels of practices (odds ratio = 4.18, *p* < 0.05).

## 4. Discussion

Based on the above results, several interesting and important points came up. Even though 93.8% of participants stated that they were aware of the recommendations regarding SARS-CoV-2, only 21.6% of participants correctly responded that antibiotics are not useful for the treatment of COVID-19 infection. This percentage is smaller than the correct response rate of 38.3% to the same question found in a similar study [36]. Moreover, 10.8% of survey respondents replied that there is a specific treatment for COVID-19. This percentage is lower than the reported 14% incorrect answer rate on a similar question in a study by Mohammed Basheeruddin Asdaq et al. However, in the above-mentioned study, the study population included non-physician staff, such as nurses and pharmacists. The physicians’ incorrect response rate was 7% [13]. Notably, 89% of participants stated that they were vaccinated against COVID-19. As seen in the study by H.C. Maltezou et al., 71.3% of healthcare professionals reported an intention to be vaccinated [37]. Moreover, a study among physicians from central Greece reported that the acceptance of vaccination against COVID-19 was estimated at 78.5% [38]. Therefore, the percentage of non-vaccination seen in primary care physicians is lower than expected. The issue of COVID-19 vaccination nevertheless remains a controversial subject for the public, and among physicians [37,39,40]. As previously mentioned, after vaccine rollout, several countries implemented some form of vaccine incentive, either as a green pass, or as a fine for non-vaccinated individuals [28,29]. A study in the elderly population in southern Italy showed that after the green pass adoption, fewer people supported the vaccine mandate [29]. Similar results were documented in Israel, where the vaccination campaign showed an early and dynamic process. When asked about the green pass, 31% of unvaccinated Israeli citizens reported that the green pass would motivate them to be vaccinated but 46% replied negatively. Moreover, rising issues regarding the debate on a mandatory option for vaccine coverage, in terms of individual autonomy and freedom limitation, have hindered more drastic action [41]. Unfortunately, due to the timing of our study (at the start of the vaccination campaign), we were not able to incorporate questions regarding a COVID-19 vaccine mandatory option.

Regarding the proper use of PPE, the most significant findings of our study are those about facemask use. Interestingly, 22.2% of participants touch the facemask while wearing it while 30.4% of physicians do not immediately dispose of the mask in a closed bin immediately after its use. The above finding suggests that 40.7% of participants reuse their facemask during the day, despite clear WHO guidelines [42]. In an Ethiopian study, the percentage of facemask re-use was 90%, whereas 60% of healthcare professionals touched their mask while wearing it [12]. This is very important, as studies associate facemask reuse with a higher chance of testing positive for COVID-19 [10]. Even though the percentage of non-compliance found in this survey is lower compared to similar studies [12], these results remain alarming.

As shown in Table 3, the mean score level of participants in the knowledge section is significantly lower in comparison to the average score levels of the other categories. Higher levels of knowledge (75th percentile and above) were displayed by 7.7% (n = 15) of physicians. Other studies examining physician knowledge with similar scoring systems have reported good knowledge (80% or better answer rate) in 53% of their participating doctors [13], with similar findings in a study from Pakistan (56% of physicians had an 85% or better correct answer rate in knowledge questions) [43]. In another study examining COVID-19 preparedness among primary healthcare workers in Greece, it was found that primary healthcare workers, with different disciplinary formations, working in rural settings were more prepared in comparison to ones working in urban areas [44]. Mixed methodology, for further research on similar topics, may offer an additional input to explain this apparent diversity.

An important finding is the higher rate of correct practices seen in the private sector (87.6) when compared to the public sector (81.9). This finding could be explained by the higher availability of healthcare materials in the private sector, which leads to better compliance with international guidelines. However, to the best of our knowledge, this finding has not been described in another KAP study. Another interesting finding presented is that physicians who contracted COVID-19 had a lower average knowledge score compared to those who did not (39.7 vs. 50.2), which was a statistically significant finding. A limited knowledge score among infected physicians may drive higher risk behaviors, reduced use of personal protective equipment, or negligence and therefore a higher risk of infection from COVID-19, as supported by an Indian study [45]. In this study, healthcare workers perceived negligence as a critical reason to be infected from COVID-19 [45]. However, a reversed way to approach these findings is that a lack of training of frontline physicians on the use of PPE is conditioned by their poor knowledge scores, leading to a higher infection risk. This description is aligned with a previously mentioned study regarding personal protection measures [11].

Finally, a hierarchical multiple logistic regression analysis of higher-level vs. lower-level practices, shown in Table 4, revealed significantly higher odds of people with high levels of attitude scores to have high practices scores. This association of positive attitudes and good practices has been described by other studies [13,21,46]. However, the strength of the association between the two varies. Indeed, several lines of evidence suggest considerable variability in the degree to which attitude is a determinant of practice [47]. This is also the case for the association between knowledge and practice. Furthermore, the results of a Greek population KAP survey showed that an adequate level of knowledge does not inevitably ensure good practice [48]. Notwithstanding these methodological considerations, an interesting finding of our study is that attitudes were the only significant predictor of proper practices related to the prevention of COVID-19. Consequently, if replicated by future research, this finding may have important policy implications for prevention. The policy implication would be to improve physicians’ attitudes towards prevention, which in turn would enhance their preventive practice.

### 4.1. Study Contributions and Implications

The present study attempted to determine the KAP of PCPs in Greece during the beginning of the COVID-19 vaccination campaign. Even though several findings in our study were interesting and significant, the authors think that the contribution of this study to the national and international literature can be summarized into two important points. Firstly, this study’s population group, namely primary care physicians, was homogeneous, since most KAP studies were either conducted in the general population or among healthcare workers in general [13,15,16,17]. It is important that the knowledge of frontline physicians is also assessed, as they may offer person-centered services. This study can be used to discuss influences that geographical or health-system-related factors may have on the above-mentioned scores. Secondly, the timing of this study, almost a year after the start of the pandemic, allowed the authors to be confident that any gaps found in the knowledge, attitudes, and practices of physicians were time-captured findings and can be useful for future health planning. What is implied by this study is that more knowledge does not necessarily mean better practices for doctors, and therefore efforts should not be solely focused on improving the knowledge of physicians but also identify ways to achieve better implementation. Finally, our study revealed a vaccination frequency that was better than expected among PCPs in the early phase of vaccination. However, direct comparisons with the vaccination rates of the general population or mixed healthcare worker groups deserve attention since data collection processes differ in terms of the time and sample [29,37,38]. From the standpoint of the primary care doctor, this can mean that a more effective and individualized approach to vaccination is required when discussing the matter with patients. Of course, the intervention of the PCP is not the only determinant of the final decision regarding vaccination, but it is a component that motivates such a decision [49].

### 4.2. Limitations

Our study had several limitations, which can affect the observed findings. Firstly, the study is limited by its cross-sectional nature because we are unable to differentiate cause and effect. Second, the results were based on answers from a self-administered questionnaire and there is a risk of information bias. Third, the survey was distributed via email, and a subscription within a single platform was a technical prerequisite. Therefore, physicians without an email address who were not subscribed were immediately excluded. Fourth, the time window to complete the survey was one month, which means that participants had a relatively short time interval to submit their answers. In relation to any selection bias, the authors consider that the emailing procedure includes risks of non-responsiveness due to e-address inactivity, low reply adherence as a tactic, or unwillingness of the respondents to communicate their responses through email. However, the response rate of our survey (38.4%) may be considered satisfactory for an electronic survey or general surveys with the participation of physicians [30,31,32]. An attempt to assess the knowledge, attitudes, and practices about COVID-19 using a simple questionnaire administered to PCPs was made during the pandemic and in the early phase of community vaccination. The lack of validated metric tools for emergency periods (pandemic), or evidence to guide the validity (of the questionnaire) and be used to estimate the sample size (power, type I-error, precision d, etc.) was and remains a realistic weakness. In addition, the authors found it challenging to assess the consistency of responses (reliability) of the participants to specific, usually changing, measures or recommendations when specific domains are considered (e.g., have the recommendations for SARS-CoV-2 changed your daily practices regarding personal protective measures in your workspace?). The authors performed post hoc analysis for sample size estimation from the knowledge score comparisons between participants who contracted COVID-19 or not (two tails, r effect size = 0.62, power > 80% and α = 0.05), and found that the minimum required sample was marginally equal to the sample recruited [50]. However, this assessment was performed a posteriori and for this reason is presented in the limitations section.

Due to the timing of our study, as mentioned, the authors were not able to include items on the mandatory vaccination option or vaccine incentives [29].

## 5. Conclusions

This is one of the few studies that has explored the knowledge, attitudes, and practices towards SARS-CoV-2 among primary care physicians in Greece, to the best of our knowledge. The vaccination rate was higher than expected during the beginning phase and without any discussion about duty obligation at that time. We found that attitudes were the only significant determinant of proper practices towards the prevention of COVID-19. This finding, if confirmed by future research, may have an important policy impact on prevention strategy planning. It is important to note that non-compliance with recommendations regarding PPE use could be partially attributed to medical supplies shortage management during an earlier phase, which has led WHO to call on industry and governments to increase manufacturing by 40% to meet rising global demand [51]. Our study findings also reveal that although primary care physicians have an adequate level of knowledge regarding the pandemic, there are certain points of uncertainty. Even though most general practitioners report that they are aware of the recommendations of the WHO, some primary care physicians do not use the available PPE properly [49], which may lead to increased physician infection rates. Further studies of this type could be performed to highlight the findings of the current survey.

## Figures and Tables

**Table 1:** General characteristics of 194 primary care physicians and residents that participated in the study.

General Characteristics		% (n)
Gender	male female	42.3 (82) 57.7 (112)
Age, years	mean ± stand. Dev. (min, max)	46.5 ± 8.0 (25, 68)
	50+	29.4 (57)
Years from residency completion	mean (median) [range]	10.4 (9.0) [0–35]
	current resident	8.8 (17)
	up to 10 years	50.5 (98)
	>10 years	40.7 (79)
Ph.D./MSc degree	no yes	66.0 (128) 34.0 (66)
Workplace	Hospital	20.6 (40)
	Health Center	36.1 (70)
	Satellite Practice	17.0 (33)
	Private Practice	26.3 (51)
During the pandemic, did you contract the novel coronavirus (SARS-CoV-2)?	No yes	89.2 (173) 10.8 (21)

In total, 92 different municipalities were reported as places of stay.

**Table 2:** Correct response rate of 194 primary care physicians and residents to questions regarding knowledge, attitudes, and daily practices during the pandemic (NOPH: National Organization for Public Health).

**Knowledge (Right Responses)**	**% (n)**	**95%Cis**
Which was your main source of information regarding SARS-CoV-2? (International Healthcare Organizations: WHO, ECDC, CDC)	36.1 (70)	29.6, 43.0
Are you aware of the WHO recommendations regarding SARS-CoV-2?	93.8 (182)	89.8, 96.6
Is there a specific treatment available for coronavirus (SARS-CoV-2)?	10.8 (21)	7.0, 15.8
Are antibiotics useful in the treatment of COVID-19 infection?	21.6 (42)	16.3, 27.8
Are the recommendations of healthcare authorities in Greece regarding coronavirus (SARS-CoV-2) adequate?	20.1 (39)	14.9, 26.2
Is coronavirus (SARS-CoV-2) sexually transmitted?	85.6 (166)	80.1, 90.0
Can coronavirus (SARS-CoV-2) be transmitted by consuming food?	15.5 (30)	10.9, 21.0
Can coronavirus (SARS-CoV-2) spread via respiratory droplets?	80.9 (157)	75.0, 86.0
Are coronavirus (SARS-CoV-2) symptoms similar to influenza?	76.8 (149)	70.5, 82.3
**Attitudes (Positive Responses)**		
Do you believe that you followed the published NOPH recommendations accurately?	92.3 (179)	87.9, 95.4
Are you vaccinated against coronavirus (SARS-CoV-2) or promptly intended to be? (n = 29 participants willing to be vaccinated whose vaccination appointment had not been scheduled yet are included)	88.7 (172)	83.6, 92.5
Have the recommendations for SARS-CoV-2 changed your daily practices regarding personal protective measures in your workspace?	95.4 (185)	91.7, 97.7
Do you advise your patients to use soap or sanitizer, and to wash their hands regularly?	95.4 (185)	91.7, 97.7
Do you advise your patients to avoid close contact with people with cough or other viral illness symptoms?	99.0 (192)	96.7, 99.8
**Practices (Right Responses)**		
Do you wash your hands with water and soap/use hand sanitizer before touching the facemask?	83.0 (161)	77.2, 87.8
Do you always check that the correct side of the facemask faces up when wearing it (where the metal strip is)?	99.5 (193)	97.6, 99.9
Do you always check that the correct side of the facemask faces outward (the colored side)?	100.0 (194)	--
Do you touch the facemask while wearing it?	77.8 (151)	71.6, 83.2
Do you dispose of the facemask in a closed bin immediately after its first use?	69.6 (135)	62.9, 75.7
Do you reuse your facemask during the day?	59.3 (115)	52.3, 66.0
Do you repeat hand hygiene practices (e.g., washing hands with soap/using hand sanitizer) after touching or disposing of the facemask?	93.3 (181)	89.1, 96.2
During the COVID-19 pandemic, are you always wearing the required personal protective equipment (e.g., facemask, gloves, gown) when in contact with patients?	89.7 (174)	84.8, 93.4
How often do you wash your hands when working?	83.5 (162)	77.8, 88.2
How often do you change examination gloves?	78.4 (152)	72.2, 83.7

**Table 3:** Score levels of knowledge, attitudes, and daily practices during the pandemic of 194 primary care physicians and residents. Scores were transformed on a 0 to 100 scale where higher indicates better knowledge, more positive attitudes, or right practices.

	Mean (Stand. Dev)	Median (Min, Max)
Knowledge (9 items/questions) higher levels (75.0+),	49.0 (15.7) 7.7% (n = 15)	44.4 (22.2, 88.9)

Attitudes (5 items/questions) higher levels (75.0+),	94.1 (11.4) 94.3% (n = 183)	100.0 (60.0, 100.0)

Practices (10 items/questions) higher levels (75.0+),	83.4 (17.9) 71.1% (n = 138)	90.0 (30.0, 100.0)


**Table 4:** Hierarchical multiple logistic regression analysis of practices’ higher levels (vs. lower) in 194 primary care physicians and residents in relation to knowledge, attitudes, and their general characteristics.

	Practices (Higher Levels vs. Lower)					
	1st Model		2nd Model		3rd Model	
	Odds Ratio	95%CI	Odds Ratio	95%CI	Odds Ratio	95%CI
Gender (females vs. males)	0.89	0.46, 1.73	0.89	0.46, 1.73	0.87	0.44, 1.72
Age (per year)	1.06	0.99, 1.14	1.06	0.99, 1.14	1.05	0.98, 1.13
Years from residency completion (per year)	0.96	0.90, 1.02	0.96	0.90, 1.02	0.96	0.90, 1.03
Ph.D./MSc degree (yes vs. no)	1.05	0.53, 2.07	1.05	0.53, 2.07	1.06	0.53, 2.13
Workplace (Private sector vs. Public sector)	1.54	0.71, 3.36	1.53	0.69, 3.35	1.45	0.65, 3.23
During the pandemic, did you contract the novel coronavirus (SARS-CoV-2)? (yes vs. no)	1.10	0.39, 3.07	1.11	0.40, 3.11	1.30	0.44, 3.85
Knowledge (Higher levels vs. lower)	--		1.10	0.33, 3.71	1.01	0.30, 3.43
Attitudes (Higher levels vs. lower)	--		--		4.18 *	1.13, 15.50
Pseudo R^2^_Nagelkerke_	0.038		0.039		0.073	

* *p* < 0.05.

## Data Availability

Not applicable.
